# Strain Acquisition Framework and Novel Bending Evaluation Formulation for Compression-Loaded Composites Using Digital Image Correlation

**DOI:** 10.3390/ma14205931

**Published:** 2021-10-09

**Authors:** Jonas J. A. D’haen, Michael May, Octavian Knoll, Stefan Kerscher, Stefan Hiermaier

**Affiliations:** 1 BMW AG, Knorrstraße 147, 80788 Munich, Germany; Octavian.Knoll@bmw.de (O.K.); Stefan.SJ.Kerscher@bmw.de (S.K.); 2 Fraunhofer Institute for High-Speed Dynamics, EMI, Ernst-Zermelo-Straße 4, 79104 Freiburg, Germany; Michael.May@emi.fraunhofer.de (M.M.); Stefan.Hiermaier@emi.fraunhofer.de (S.H.)

**Keywords:** mechanical properties, mechanical testing, digital image correlation, composite material characterization

## Abstract

Consistent and reproducible data are key for material characterization. This work presents digital image correlation (DIC) strain acquisition guidelines for compression-loaded carbon fiber composites. Additionally, a novel bending criterion is formulated which builds up on the DIC strain data so that it is able to completely replace state-of-the-art tactile strain devices. These guidelines are derived from a custom test setup that simultaneously investigates the front and side view of the specimen. They reflect both an observation and post-processing standpoint. It is found that the DIC-based strain progress matches closely with state-of-the-art strain gauges up to failure initiation. The new bending evaluation criterion allows the bending state—and therefore, the validity of the compression test—to be monitored analogously to the methodology defined in the standards. Furthermore, the new bending criterion eliminates a specific bending mode, caused by an offset of clamps, which cannot be detected by the traditional strain gauge-based monitoring approach.

## 1. Introduction

Compression loading is the most important load case for structures designed to withstand a crash. Understanding the behavior during this loading is crucial in designing such structures. Several research groups have therefore worked on establishing knowledge on compressive loading behavior [1,2,3,4,5,6].

The optimization of the load introduction into the specimen has been given major attention as deviations from the perfect loading conditions result in bending of the specimen and therefore undesired failure modes. Either end loading, shear loading, or moment loading are used to load the specimen [7,8]. One of the first advanced load introduction methods must have been the Celanese fixture developed by Park [9]. Hofer and Rao presented an improved version eliminating the wedge seating problems induced by the conical wedges of the Celanese fixture [10], which is also known under the name “Illinois Institute of Technology Research Institute”(IITRI). An extensive comparison between the Celanese fixture, end loading rig from Wyoming Test Fixtures, and IITRI clamp has been performed by Sendeckyj et al. [11]. The IITRI reported the best strength and lowest scatter in modulus measurements [11,12]. Due to the limited available space and extreme behavior after failure, Sendeckyj recommends using strain gauges instead of an extensometer [11].

Around the same time that Hsiao showed his fixture for thick laminates [7], Adams and Welsh developed a combined loading compression (CLC) test method based on Irion’s end loading fixture [13,14]. The advantage of a CLC fixture is that the load is introduced using shear and end loading on the specimen. Adams and Welsh conclude that the fixture is easy to use, and that consistent measurement data are obtained. Nowadays, compression tests are very often performed using a CLC fixture. Guidelines for testing can be found in the ASTM D6641 [15], ASTM D695 [16], DIN EN ISO 14126 [17], or the work by Adsit [18].

In standard analyses of compression tests, the strain is captured using two strain gauges bonded to opposing faces of the coupon. However, after first ply failure, the strain recorded by the strain gauges is not representative for the coupon anymore. For analyses of unidirectional composite materials, where sudden, catastrophic failure occurs after damage initiation, this seems to be acceptable. However, in real life applications, multidirectional laminates are used. For multidirectional laminates, the damage and failure sequence are significantly different from unidirectional composite materials as distinct post-failure behavior can be observed after first ply failure, which cannot be analyzed with strain gauge-based measurement approaches. As the field on how to measure strain after failure initiation is yet uncovered, this research will contribute to setting up conventions for measuring strain after failure initiation. The applied strain measuring technique in this work is digital image correlation (DIC), a technique developed in the 1980s and described in multiple publications [19,20,21,22,23,24]. DIC has proven to be an efficient method to analyze composite materials [19,25,26,27,28,29]. The literature review revealed a gap in the application of DIC on compression-loaded composites. For example, there is no efficient possibility to monitor the bending state using DIC information. The DIC strain measurement technique will be applied on top of the specifications described in the DIN EN ISO 14126 testing standards. The goal of this paper is to provide a DIC strain acquisition framework for compression-loaded composites. Furthermore, it aims to define a new DIC-based bending formulation similar to the one defined in material testing standards [30,31]. The consecutive intention is to make advancements in the material characterization process by standardizing strain measurements that can be used to record strain evolution during damage propagation. These topics will be tackled in Section 2 followed by the discussion of the results in Section 3. Section 4 presents the novel bending criterion before the article finalizes with the conclusion in Section 5.

## 2. Test Setup and Strain Measurement Method

**Figure 1:**
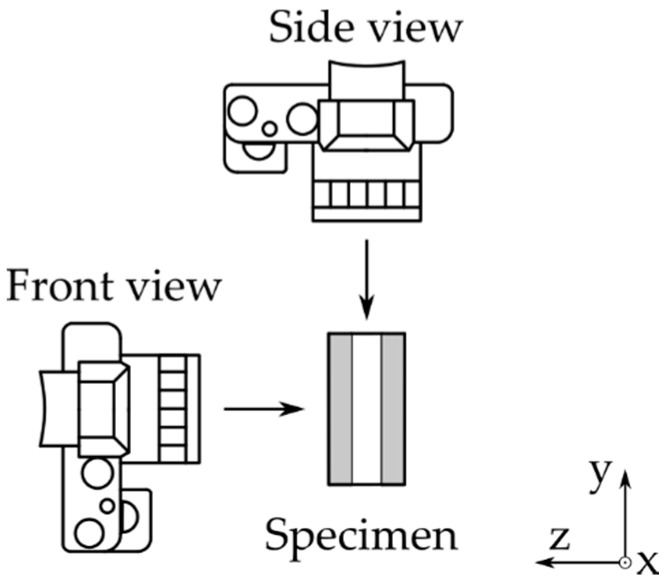
DIC camera setup seen from the top side.

Conventionally, strain gauges are applied on the front and back side of the compression specimen. For DIC measurements, it is possible to measure strain at all sides of the specimen. In order to determine the most favorable side, an investigation was performed. This results in a test setup with two cameras observing the front and side view of the specimen as the symmetrical sides are considered to be the same. A top view of the test setup for this investigation is shown in Figure 1.

**Figure 2:**
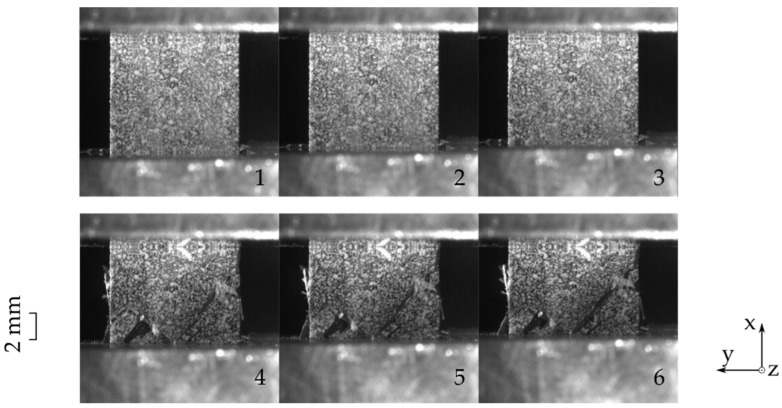
Progressive failure in a 0° loaded [45,-45,0]_s_ laminate observed from the front view (**1**–**6** chronologically obtained images).

The composite used in this work was a carbon epoxy laminate with a stacking sequence of [45,-45,0]_s_, manufactured using the resin transfer molding (RTM) production process. The carbon fiber layers provided by SGL are relatively thick due to its constellation of 50K fibers per roving. The specimens were extracted using waterjet cutting from larger plate substrates. Clamping of the specimen was undertaken using a combined loading rig (HCCF Zwick Roell) and compressed using a Z250 Zwick Roell machine. The HCCF rig allows for a simultaneous observation of the front and side view. Figure 2 shows six front view images from the composite loaded in 0° direction. The images were chronologically extracted from the test sequence, with the last three images showing detachment of the outer layer towards the camera. This detachment is also known as delamination and leads to, when looking at the front, strain data from the disjoined layer(s) instead of the entire specimen. In this particular case, strain gauges placed on the surface of the specimen do not provide any meaningful information anymore.

**Figure 3:**
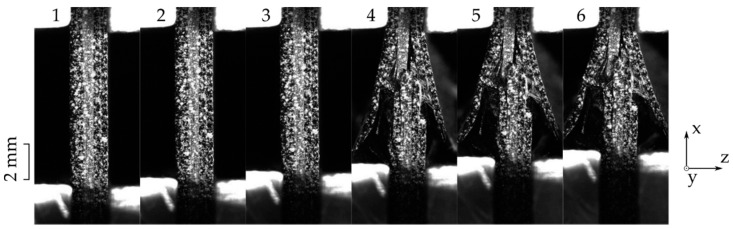
Progressive failure in a 0° loaded [45,-45,0]_s_ laminate observed from the side view (**1**–**6** chronologically obtained images).

The side view images for the same test are shown in Figure 3. This perspective allows an investigation of all layers of the laminate. For this layup, it is obvious that the middle two 0° layers carry the highest load. Once they break, a high deformation and stiffness degradation takes place. Looking closer at the third image, the middle two layers are broken. Similar to the front view sequence, delamination can be observed in the third image. From the side view, it becomes clear that this delamination occurs on both the front and the back side, supporting the previously described weakness.

### Strain Post-Processing Techniques

**Figure 4:**
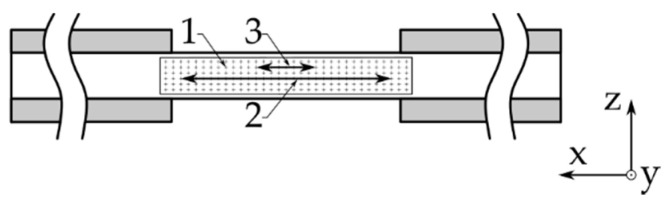
The investigated strain post-processing techniques applied to a compression specimen: **1**. strain field measurement, **2**. long extensometer, **3**. short extensometer.

An additional interest of the work lies in the strain behavior after initial failure (AIF). In order to capture this information accurately, three DIC strain post-processing methods were investigated. These three methods are strain field measurement (1), long extensometer (2), and short extensometer (3), which are all shown in Figure 4. Localized options, smaller than strain gauges, were not considered due to the noise on the localized signal and due to the fact that it measures localized material effects, which were not of interest.

These three approaches can be analyzed on a single dataset as the post-processing is decoupled from the test itself. The software used to post-process the data was ARAMIS [30]. The three methods reported very similar results up to initial failure, as a difference could only be observed after passing this point. The investigation shows that the long extensometer reports results with the smallest scatter and marginal manual intervention. The short extensometer on the other hand requires too much manual interaction as it needs to be placed over the crack. Strain field measurement fails to record the degradation because the direct area around the crack fails to provide measurement information due to its grave state change. Other data points obtained by the strain field report an unloaded condition as the material relaxes in the non-crack area. Therefore, the long extensometer approach will be used as the default strain measurement technique throughout this work.

## 3. Results

**Figure 5:**
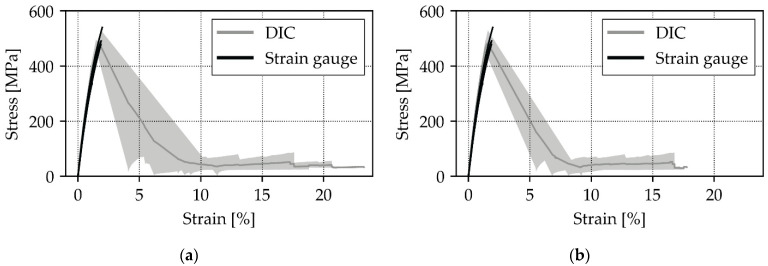
(**a**) Front view DIC and strain gauge comparison for a 0° oriented compression-loaded laminate.; (**b**) side view DIC and strain gauge comparison for a 0° oriented compression-loaded laminate.

Comparing the collected DIC results with state-of-the-art strain gauge results leads to Figure 5a (front view) and Figure 5b (side view). The visualization of DIC data was performed by plotting a light gray area that spans between the minimum and maximum value of the tested specimen, whereas the dark gray line represents the reported mean value. Strain gauge results are plotted individually for a simple distinction with the DIC data. The front and side view DIC data are compared to the same strain gauge data in order to allow a straight-forward comparison. The results up to failure are in good agreement and variation of the two measurements are found to be in a similar corridor. Furthermore, the variance of the strain gauge-based and DIC-based modulus is within the typical expected range. Strain gauges fail to record data after the maximum stress point. Subsequently to this stage, both measurement techniques drift apart. The applied force drops, and the strain increases considerably. This effect happens so fast that the DIC system only manages to record one data point after the brisk deformation. The reason for this sudden change is the failure of the main load carrying 0° layers which are known to fail abruptly. Comparing the front and side view, it can be observed that the side view reports the most consistent values as the scattering range is found to be smaller. The front view shows more data points at large strains, which is caused by delayed stopping of the front view camera.

### Front or Side View, That Is the Question

Previously presented results only show a small difference between the front and the side view. However, there are multiple criteria that should be considered when deciding on the camera’s point of view. The following list elaborates on them:

- Strain results from the side view are found to be more consistent due to a smaller scatter regime in comparison to the front view results, as shown in Figure 5a,b.
- In case of delaminated outer layers, DIC measurements from the front view report local strain values instead of the global laminate behavior.
- Figure 2 in comparison to Figure 3 shows that side view figures reveal additional information on the arising failure modes. It can therefore be said that the side view allows a better failure mode recognition.
- Bending is an unfavorable load state which leads to early failure. This state should therefore be monitored so that faulty loaded measurements can be scrapped. For this case, the side view is the preferred viewing point as it allows for a better out of plane deformation observation.

This overview underlines the preference of side view assessment over front view images and is there with the recommended observation direction.

## 4. Novel Bending Criterion

Bending in a compression specimen leads to an inhomogeneous stress state and could therefore result in an unfavorable early failure. The testing standard DIN EN ISO 14126 prescribed the observation of the bending state (also known as bending evaluation) using Equation (1). This equation is based on the strain input from the front and backside of the specimen.

(1) $$B_{y}=\frac{\varepsilon_{1}-\varepsilon_{2}}{\varepsilon_{1}+\varepsilon_{2}}\;x\;100,$$

where $B_{y}$ is the bending state, $\varepsilon_{1}$ is the frontside strain, and $\varepsilon_{2}$ is the backside strain. These data, however, are by default not present from side view DIC measurements. Tracking the strain at the edge of the specimen is considered inadequate as these measuring points contain a lot of noise. A new approach that is compatible with side view DIC measurements should therefore be considered before a complete transition towards a DIC only strain measurement setup can be carried out. This transition is important as it would significantly reduce specimen preparation time and reduce the complexity of the test setup.

**Figure 6:**
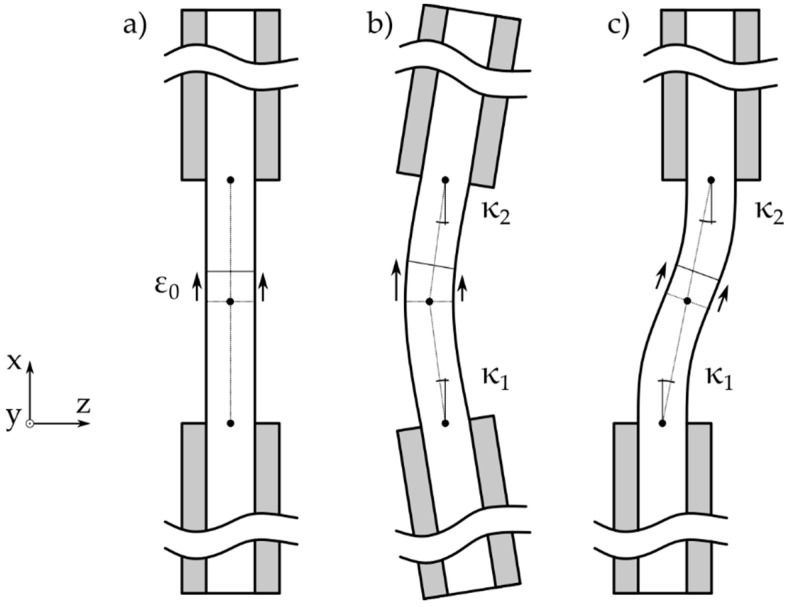
Three possible bending states in a compression specimen: (**a**) ideal load introduction; (**b**) inclined clamps; and (**c**) offset clamps.

Figure 6 shows three possible bending states the compression specimen could be subjected to. Additionally, $\varepsilon_{0}$ as pure compression strain, $\kappa_{1}$ as bending angle 1, and $\kappa_{2}$ as bending angle 2 are visualized in Figure 6. State (a) shows a perfectly loaded specimen with a constant strain distribution over the thickness. In the middle, state (b), shows inclined loading rig clamps leading to a non-constant strain distribution at the center of the specimen. This is a typical suspect that is scrapped due to the 10% maximum allowable bending state (Equation (1)). The third state (c) is achieved with an offset between the upper and the lower clamp. This misalignment results in an inhomogeneous stress state in the specimen; however, the strain at the center of the specimen would be constant. This means that this bending state is not detected by the classical bending evaluation equation, even though it is very likely that the specimen would fail early. For the side view DIC recording, it was proposed to evaluate the bending state by measuring the out of plane deformation angle of the lower and the upper side of the specimen. These angles, $\kappa_{1}$ and $\kappa_{2}$, are visualized in Figure 6. The rotation of these angles should be observed during testing as an increase would suggest a faulty load state in the specimen. $\kappa_{1}$ and $\kappa_{2}$ should be calculated against the reference frame instead of the vertical axis in order to minimize the positioning influence of the points. It was decided to use −0.1° and 0.1° as boundaries for angle $\kappa_{1}$ and $\kappa_{2}$ as they result in a close match to Equation (1). The new formulation is shown in Equation (2):

(2) $$\begin{array}{c} -0.1^{{}^{\circ}}<\kappa_{1}<0.1^{{}^{\circ}}\\ -0.1^{{}^{\circ}}<\kappa_{2}<0.1^{{}^{\circ}} \end{array}$$

A test series has been performed to compare the integrity of the new bending criterion with the strain gauge-based one. In order to have a fair comparison, special specimens have been crafted that enable simultaneous DIC and strain gauge measurements.

**Figure 7:**
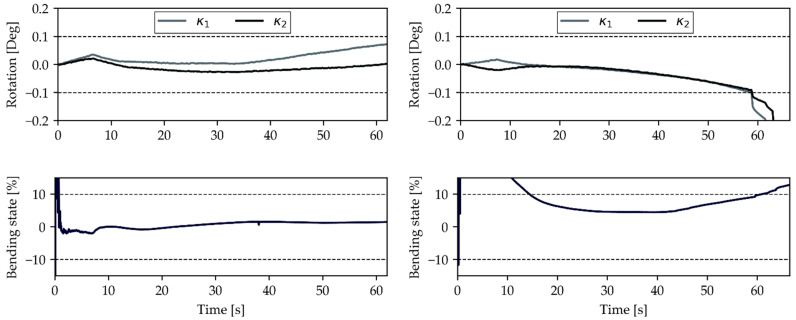
Comparison of the newly proposed bending criterion (**upper plot**) with the state-of-the-art (**lower plot**) on a UD [0]_6_ laminate in 0° direction. Left side shows specimen 1, the right side presents data obtained from specimen 4 of the test series.

A critical look at the plots from specimen 1 (Figure 7) shows that the bending evaluation exceeds the prescribed limit. High initial bending percentages are a recurring problem for the strain gauge evaluation. This can be explained by the nature of Equation (1), as $\varepsilon_{1}=-\varepsilon_{2}$ would lead to an infinitely high percentage of bending, possibly caused by noise or realignment in the fixture. The response of specimen 1, visualized in the previous figure, shows that this measurement stays within its boundaries and therefore does not need to be dismissed from the test series. Specimen 4 shows the opposite scenario as it exceeds the defined boundaries for both criteria after 60 s. Apart from this initial discrepancy, it is safe to say that a similar trend exists between both criteria. The bending evaluation has been considered the last hurdle before a full practical transition towards DIC strain measurements could be made. This is because mounting two DIC systems, one on each side, to check the bending state described in the testing standard is simply too impractical, inefficient, and expensive. This new criterion, which only requires one camera monitoring the edge of the specimen, therefore allows for the full switch towards DIC measurements without losing the established plausibility check.

## 5. Conclusions

This work demonstrates best practices for the application of DIC on carbon fiber laminate compression tests. These recommendations have been extracted from a custom test setup that simultaneously recorded DIC data from the front and side view of the specimen. From this comparison, the following guidelines are extracted:

- DIC should be performed on the side view of the specimen because results are more consistent, and delamination of the outer layer cannot block the strain measurement. Furthermore, failure mode assessment and bending state evaluation work better when looking at the side of the specimen.
- A virtual long extensometer results in the preferred approach to extract strain information from the DIC data, as it enables the strain acquisition after first ply failure.

A new, angular rotation-based approach facilitates the evaluation of bending, and therefore the validity of the test, from the optical recordings without the need for additional strain gauges applied to the specimen. It was demonstrated that obtained results are in line with traditional strain gauge findings and allow for better quality control as it is able to detect an additional bending mode which cannot be detected by the traditional bending formulation. The new criterion allows for a complete switch to DIC without sacrificing state-of-the-art validity checks.

## Data Availability

The data presented in this study are available on request from the corresponding author.

## References

[1] Shuart M.J. (1989). Failure of compression-loaded multidirectional composite laminates. AIAA J..

[2] Kindervater C.H. (1986). Compression and crush energy absorption behaviour of composite laminates. Ed. Phys..

[3] Kyriakides S., Arseculeratne R., Perry E.J., Liechti K.M. (1995). On the compressive failure of fiber reinforced composites. Int. J. Solids Struct..

[4] Vogler T.J., Kyriakides S. (1997). Initiation and axial propagation of kink bands in fiber composites. Acta Mater..

[5] Soutis C., Curtis P.T. (2000). A method for predicting the fracture toughness of CFRP laminates failing by fibre microbuckling. Compos. Part A Appl. Sci. Manuf..

[6] Camponeschi E.T., O’Brien T.K. (1991). Compression of composite materials: A review. Composite Materials: Fatigue and Fracture.

[7] Hsiao H.M., Daniel I.M., Wooh S.C. (1995). A new compression test method for thick composites. J. Compos. Mater..

[8] Wegner P.M., Adams D.F. (2000). Verification of the Combined Load Compression (CLC) Test Method.

[9] Park I.K. Tensile and Compressive Test Methods for High-Modulus Graphite Fibre-Reinforced Composites. Proceedings of the International Conference on Carbon Fibres, Their Composites and Applications.

[10] Hofer K.E., Rao P.N. (1977). A new static compression fixture for advanced composite materials. J. Test. Eval..

[11] Sendeckyj G.P., Wang S.S., Johnson W.S., Stinchcomb W.W., Pagano N.J., Berg J.S., Adams D.F. (1989). An evaluation of composite material compression test methods. J. Compos. Technol. Res..

[12] Tan S.C. (1992). Stress analysis and the testing of celanese and IITRI compression specimens. Compos. Sci. Technol..

[13] Irion M.N., Adams D.F. (1981). Compression creep testing of unidirectional composite materials. Composites.

[14] Adams D.F., Welsh J.S. (1997). The wyoming combined loading compression (CLC) test method. J. Compos. Technol. Res..

[15] ASTM (2009). Standard Test Method for Compressive Properties of Polymer Matrix Composite Materials Using a Combined Loading Compression Test Fixture.

[16] ASTM (2002). Standard Test Method for Compressive Properties of Rigid Plastics.

[17] DIN Deutsches Institut für Normunge (2000). V. Bestimmung Der Druckeigenschaften in Der Laminatebene.

[18] Adsit N., Chait R., Papirno R. (1983). Compression Testing of Graphite/Epoxy. Proceedings of the Symposium on Compression Testing of Homogeneous Materials and Composites.

[19] Ivanov D., Ivanov S., Lomov S., Verpoest I. (2009). Strain mapping analysis of textile composites. Opt. Lasers Eng..

[20] Wang C.C.-B., Chahine N.O., Hung C.T., Ateshian G.A. (2003). Optical determination of anisotropic material properties of bovine articular cartilage in compression. J. Biomech..

[21] Jerabek M., Major Z., Lang R.W. (2010). Strain determination of polymeric materials using digital image correlation. Polym. Test..

[22] Sánchez-Arévalo F.M., Pulos G. (2008). Use of digital image correlation to determine the mechanical behavior of materials. Mater. Charact..

[23] Taher S.T., Thomsen O.T., Dulieu-Barton J.M., Zhang S. (2012). Determination of mechanical properties of PVC foam using a modified arcan fixture. Compos. Part A Appl. Sci. Manuf..

[24] Funari M.F., Spadea S., Lonetti P., Lourenço P.B. (2021). On the elastic and mixed-mode fracture properties of pvc foam. Theor. Appl. Fract. Mech..

[25] Orell O., Vuorinen J., Jokinen J., Kettunen H., Hytönen P., Turunen J., Kanerva M. (2018). Characterization of elastic constants of anisotropic composites in compression using digital image correlation. Compos. Struct..

[26] Potluri P., Young R.J., Rashed K., Manan A., Shyng Y.T. (2009). Meso-scale strain mapping in UD woven composites. Compos. Part A Appl. Sci. Manuf..

[27] Anzelotti G., Nicoletto G., Riva E. (2008). Mesomechanic strain analysis of twill-weave composite lamina under unidirectional in-plane tension. Compos. Part A Appl. Sci. Manuf..

[28] Daggumati S., Voet E., Van Paepegem W., Degrieck J., Xu J., Lomov S.V., Verpoest I. (2011). Local strain in a 5-harness satin weave composite under static tension: Part I–experimental analysis. Compos. Sci. Technol..

[29] Flament C., Salvia M., Berthel B., Crosland G. (2016). Local Strain and damage measurements on a composite with digital image correlation and acoustic emission. J. Compos. Mater..

[30] Junginger M. (2002). Charakterisierung Und Modellierung Unverstärkter Thermoplastischer Kunststoffe Zur Numerischen Simulation von Crashvorgängen. Ph.D. Thesis.

[31] Krivachy R. (2007). Charakterisierung Und Modellierung Kurzfaserverstärkter Thermoplastischer Kunststoffe Zur Numerischen Simulation von Crashvorgängen. Ph.D. Thesis.

